# Genetic Association Between Alzheimer’s Disease Risk Variant of the *PICALM* Gene and EEG Functional Connectivity in Non-demented Adults

**DOI:** 10.3389/fnins.2020.00324

**Published:** 2020-04-16

**Authors:** Natalya Ponomareva, Tatiana Andreeva, Maria Protasova, Rodion Konovalov, Marina Krotenkova, Daria Malina, Andrey Mitrofanov, Vitaly Fokin, Sergey Illarioshkin, Evgeny Rogaev

**Affiliations:** ^1^Research Center of Neurology, Russian Academy of Sciences, Moscow, Russia; ^2^Laboratory of Evolutionary Genomics, Department of Human Genetics and Genomics, Vavilov Institute of General Genetics, Russian Academy of Sciences, Moscow, Russia; ^3^Center for Genetics and Genetic Technologies, Faculty of Biology, Lomonosov Moscow State University, Moscow, Russia; ^4^Research Center of Mental Health, Russian Academy of Medical Sciences, Moscow, Russia; ^5^Department of Psychiatry, University of Massachusetts Medical School, Worcester, MA, United States; ^6^Sirius University of Science and Technology, Sochi, Russia

**Keywords:** *PICALM* genotype, EEG connectivity, alpha rhythm, Alzheimer’s disease, genetic predisposition

## Abstract

Genome wide association studies (GWAS) have identified and validated the association of the *PICALM* genotype with Alzheimer’s disease (AD). The *PICALM* rs3851179 *A* allele is thought to have a protective effect, whereas the *G* allele appears to confer risk for AD. The influence of the *PICALM* genotype on brain functional connectivity in non-demented subjects remains largely unknown. We examined the association of the *PICALM* rs3851179 genotype with the characteristics of lagged linear connectivity (LLC) of resting EEG sources in 104 non-demented adults younger than 60 years of age. The EEG analysis was performed using exact low-resolution brain electromagnetic tomography (eLORETA) freeware (Pascual-Marqui et al., 2011). We found that the carriers of the *A PICALM* allele (*PICALM AA and AG* genotypes) had higher widespread interhemispheric LLC of alpha sources compared to the carriers of the *GG PICALM* allele. An exploratory correlation analysis showed a moderate positive association between the alpha LLC interhemispheric characteristics and the corpus callosum size and between the alpha interhemispheric LLC characteristics and the Luria word memory scores. These results suggest that the *PICALM* rs3851179 *A* allele provides protection against cognitive decline by facilitating neurophysiological reserve capacities in non-demented adults. In contrast, lower functional connectivity in carriers of the AD risk variant, *PICALM GG*, suggests early functional alterations in alpha rhythm networks.

## Introduction

Alzheimer’s disease (AD) is a devastating neurodegenerative disorder characterized by progressive impairment of memory and other cognitive functions. AD is the most common form of dementia, affecting up to 38% of people over 85 years of age (Alzheimer’s Association, 2017).

The greatest known risk factors for AD are genetic predisposition and aging. Early onset AD (EOAD) develops before the age of 65 years. EOAD is caused by mutations in the amyloid precursor protein (*APP*), presenilin-1 (*PRES-1*), and presenilin-2 (*PRES-2*) genes (Goate et al., 1991; Levy-Lahad et al., 1995; Rogaev et al., 1995; Sherrington et al., 1995).

For late onset AD (LOAD), with an age of onset older than 65 years, polymorphism of the apolipoprotein E (ApoE) gene is the most prevalent genetic risk factor (Saunders et al., 1993). Genome-wide association studies (GWAS) have identified more than 20 novel genetic risk variants associated with AD (Harold et al., 2009; Lambert et al., 2009). The phosphatidylinositol clathrin assembly lymphoid-myeloid leukaemia (*PICALM*, chrm 11q14) gene is considered to be one of the top six most prevalent genetic risk factors for AD according to data in the AlzGene database^1^.

Investigation of the impact of the genes identified in association studies on the pathogenesis of neurodegenerative diseases could potentially contribute to earlier diagnosis and development of personalized prevention strategies (Rogaev et al., 1995; Illarioshkin et al., 2004; Xu et al., 2015). *PICALM* is a 112 kb gene located on chromosome 11q14. This gene encodes an accessory adaptor protein involved in clathrin-mediated endocytosis (CME), by which cells absorb extracellular molecules (Moshkanbaryans et al., 2014). *PICALM* has been shown to contribute to AD pathogenesis through several pathways, including Aβ production, impaired Aβ and tau protein clearance, and synaptic function (Xu et al., 2015, review). The role of *PICALM* in AD development remains incompletely understood.

In addition to analyzing the genetic effects of metabolic signaling pathways at the cellular level, the genetic effects on AD pathogenesis can be revealed in vivo in the human brain by neurophysiological and neuroimaging methods.

Electroencephalography (EEG), magnetoencephalography (MEG) and functional magnetic resonance imaging (MRI) methods have provided evidence of the progressive disconnection of brain networks as a key factor in cognitive impairment in AD (Filippini et al., 2011; Canuet et al., 2012; Hata et al., 2016; Núñez et al., 2019). Resting−state functional MRI (rs-fMRI), EEG (rsEEG) and (rsMEG) detect functional connections in the brain as synchronized activity between brain regions in the absence of a task (Gómez et al., 2009; Binnewijzend et al., 2012). Functional EEG and MEG connectivity is estimated in time and in frequency domains using linear (e.g., correlation, coherence) and non-linear (e.g., lagged phase synchronization, mutual information) methods (Sakkalis, 2011; Núñez et al., 2019). The reduction of functional connectivity assessed by these methods was shown to be related to the severity of dementia in AD patients (Babiloni et al., 2018). A less pronounced decrease in functional connectivity was observed in patients with amnestic mild cognitive impairment (aMCI), which is, in most cases, a prodromal stage of AD. The analysis of resting EEG connectivity was demonstrated to be useful in estimating the risk of conversion from MCI to AD (Vecchio et al., 2018).

Neurophysiological and neuroimaging studies have revealed the effect of genetic risk factors for AD on brain functional connectivity in clinical and even preclinical stages of the disease. Several studies have demonstrated an association between EEG characteristics of functional connectivity and the AD risk variant, *ApoE E4+.* Functional network disruption in patients with early AD carrying the *ApoE E4* allele was characterized by decreased interhemispheric alpha2 connectivity between the frontal and parieto-temporal areas compared with noncarriers (Canuet et al., 2012). An EEG study by Jelic et al. (1997) using classical coherence analysis demonstrated that AD patients carrying the homozygous *ApoE E4* genotype had reduced connectivity in temporal and parietal regions. Elderly subjects without dementia carrying the *ApoE E4* allele exhibited a decrease in functional connectivity within the structures of the default mode network (DMN), which participates in various cognitive functions and is preferentially affected in AD (Lu et al., 2017). Abnormal characteristics of fMRI and MEG connectivity were recently identified in younger subjects carrying the *ApoE E4*+ genotype (Koelewijn et al., 2019).

The effect of *PICALM* genotype on functional connectivity in brain networks, in particular DMN, was found in aMCI patients and in healthy subjects in fMRI studies (Zhang et al., 2015; Sun et al., 2017). A decrease in hippocampal functional connectivity assessed by fMRI, was revealed in younger carriers of the *PICALM rs*3851179 *GG* genotype (Zhang et al., 2015). The effect of the *PICALM* genotype on EEG connectivity was not previously investigated.

We suggested that the EEG connectivity would differ depending on *PICALM* polymorphism, even in clinically healthy people. To test this hypothesis we applied exact low-resolution electromagnetic tomography (eLORETA) freeware, which is widely used to study EEG alterations in AD, aMCI and other neurodegenerative disorders. eLORETA produces a low-resolution estimation of sources of EEG signals using the inverse solution method. eLORETA lagged linear connectivity (LLC) provides linear measurements of the interdependence of cortical source activation estimated from scalp EEG rhythms (Pascual-Marqui et al., 2011). LLC characteristics of EEG connectivity are not affected by the instantaneous propagation of neural ionic currents that are partly due to head volume conduction effects. The LLC of posterior alpha sources was found to be decreased in patients with aMCI compared to normal controls (Babiloni et al., 2018).

Anatomical connections between brain structures provide the structural basis for functional connectivity. The corpus callosum (CC) is the major commissure connecting the two hemispheres. The CC mid-sagittal cross-sectional area has been shown to reflect atrophy of the brain in AD (Bachman et al., 2014). Associations between CC deficits and memory impairment have been found in AD (Parra et al., 2015). aMCI was associated with relatively less severe CC degeneration (Lee et al., 2016). CC dysfunction may contribute to network dysconnectivity by disrupting inter-hemispheric information transfer, leading to memory impairment (Hinkley et al., 2012). It is of interest to clarify the structural basis of functional connectivity in the subjects with different *PICALM* genotypes and its influence on cognitive functions.

The present study aimed to determine whether, in non-demented subjects younger than 60 years of age, polymorphism rs3851179 of the *PICALM* genotype is associated with the LLC characteristics of rsEEG cortical sources assessed by eLORETA. We also analyzed the association of the *PICALM* genotype with CC size, the correlation between LLC characteristics and CC size, and the correlations between LLC and CC size with cognitive performance scores.

## Materials and Methods

### Participants

The enrolled cohort included 104 non-demented volunteers (31 men and 73 women, age range 19–59 years).

The subjects were of Russian descent and were from Moscow and the surrounding region. The participants underwent a neurological examination and cognitive screening. The recruited subjects were free of dementia and other medical, psychiatric, and neurological conditions. The exclusion criteria included a history of neurological and psychiatric diseases, any type of memory impairment, signs of clinical depression or anxiety, physical brain injury, or other medical conditions (e.g., hypertension, diabetes, cardiac disease, or thyroid disease) or a personal history of drug or alcohol addiction.

The subjects were evaluated with the Mini-Mental State Examination (MMSE) and Clinical Dementia Rating (CDR) scale (Hughes et al., 1982). Only subjects with MMSE scores of 28 or higher and CDR scores of 0 were included in the study. All subjects were right-handed.

Written informed consent was obtained from all the participants. The experimental protocol for this study was approved by the local Ethics Committee. *ApoE* genotyping was performed for all participants, and the effect of *ApoE* genotype on EEG characteristics was statistically controlled.

All subjects were divided into subgroups according to the *PICALM (PICALM AA&AG* and *PICALM GG*) polymorphisms. The *PICALM AA&AG* group included subjects with the homozygous *PICALM AA* (18 individuals) or heterozygous *PICALM AG* (48 individuals) genotypes. The *PICALM GG* group consisted of 36 subjects with the homozygous *PICALM GG* genotype. Table 1 shows the demographic and cognitive characteristics of the participants. There were no differences in age or sex between the *PICALM GG* and *PICALM AA&AG* subgroups (*p >* 0.05). The *PICALM GG* and *PICALM AA&AG* genotype carriers had comparable scores on all cognitive tests (Table 1).

**TABLE 1:** Demographic and psychometric characteristics of participants.

	*PICALM AA&AG*	*PICALM GG*	*p* values
*N*	66	38	–
Age range	19–59	19–59	–
Age (years)	41.0+1.4	43.1+2.1	0.37
Sex (men/women)	20/46	11/27	0.88
Education (years)	15.2+0.2	14.9+0.1	0.30
*ApoE E4-/ApoE E4*+	42/24	27/11	0.29
MMSE	29.4+0.1	29.2+0.1	0.12
COWAT (words)	51.4+1.6	47.6+2.3	0.17
LMWT	6.2+0.2	6.0+0.2	0.47
SST errors (number)	0.6+0.1	1.1+0.2	0.08

*The data are presented as the means and standard errors.MMSE, Mini-Mental State Examination; LMWT, Luria memory words test; COWAT, controlled oral word association test; SST, serial sevens subtraction test.*

### EEG Recording and Data Acquisition

The registration and evaluation of EEG was carried out in accordance with the IPEG guidelines (Versavel et al., 1995; Jobert et al., 2012). All recordings were obtained in the afternoon from 3 to 4 pm. EEGs were recorded for 4 min during resting state with the subjects sitting comfortably in a chair. The subjects were asked to close their eyes and relax but to stay awake during the recording. To maintain a constant level of vigilance, an experimenter monitored the subject and the EEG traces online and verbally alerted the subject any time there were signs of behavioral and/or EEG drowsiness.

The EEGs were recorded on a Nihon Kohden 4217 G EEG (Japan) using a time constant of 0.3 s. The high frequency cut-off was 45 Hz. The 16 Ag/AgCl electrodes were placed according to the international 10–20 system at the O2, O1, P4, P3, C4, C3, F4, F3, Fp2, Fp1, T6, T5, T4, T3, F8, and F7 positions. Linked ears served as the reference. The electrode impedance did not exceed 10 kΩ. During the recordings, 180 s of EEG at rest was simultaneously sampled at 256 Hz per channel and stored on a computer for further offline analysis. The EEG was reviewed visually for artifacts, which were eliminated from the subsequent analysis. After the artifacts were eliminated, 150-s segments of the resting EEG were selected for further analysis. Frequencies below 2 Hz and above 35 Hz were eliminated using digital filtering.

### Estimation of Functional Connectivity of rsEEG Cortical Sources

The eLORETA freeware was used to estimate the lagged linear connectivity from the rsEEG rhythms (LLC, Pascual-Marqui, 2007b). LLC provides linear solutions of statistical interdependence of pairs of eLORETA cortical source activation estimated from rsEEG rhythms at a given frequency. eLORETA provides LLC solutions between all the combinations of voxels in the cortical source space of regions of interest (ROIs) (Pascual-Marqui, 2007a; Pascual-Marqui et al., 2011).

LLCs are estimated by removing the zero-lag instantaneous phase interactions between the EEG sources, which may be affected by instantaneous physical propagation of neural ionic currents due to head volume conductor effects (Pascual-Marqui, 2007b). LLC solutions also mitigate the “common-drive/source” effect of a ‘third” source by taking into account the measures of interdependence among rsEEG time series.

For each subject and frequency band (delta, theta, alpha, beta1, beta2, and gamma), LLC solutions were computed for 16 ROIs located in the same cortical areas as the electrodes (O2, O1, P4, P3, C4, C3, F4, F3, Fp2, Fp1, T6, T5, T4, T3, F8, and F7). LLC solutions were computed for all voxels of a particular ROI in the left and right hemispheres for each frequency band of interest.

To determine the cortical ROIs, eLORETA defined the MNI coordinates of the cortical voxels underlying the electrode sites. Detailed information on the eLORETA connectivity algorithm has been previously published (Pascual-Marqui et al., 2011; Babiloni et al., 2018). The 16 cortical ROIs determined by eLORETA are shown in Table 2.

**TABLE 2:** Sixteen cerebral regions of interest (ROI) determined by eLORETA.

		ROI centroid MNI coordinates		
		
Anatomical regions	Brodmann area	*x*	*y*	*z*
Right occipital	17,18,19	26.89	–114.68	9.45
Left occipital	17,18,19	–28.98	–114.52	9.67
Right parietal	7,3,9,40	53.51	–80.13	59.40
Left parietal	7,3,9,40	–55.07	–80.11	59.44
Right precentral/postcentral	1,2,3,4,6,	66.50	–12.80	65.11
Left precentral/postcentral	1,2,3,4,6,	–65.57	–13.25	64.98
Right frontal	9,46	50.38	51.84	41.33
Left frontal	9,46	–48.05	51.87	39.87
Right frontopolar	10,11	29.41	83.74	–10.04
Left frontopolar	10,11	–26.81	84.06	–10.56
Right posterior temporal	19,22,37	71.10	–75.17	–3.69
Left posterior temporal	19,22,37	–71.46	–75.17	–3.70
Right temporal	21,22,42	84.44	–16.65	–11.79
Left temporal	21,22,42	–83.36	–16.52	–12.65
Right IFG/ATC	45,47,38	68.71	41.16	–15.31
Left IFG/ATC	45,47,38	–66.99	41.69	–15.96

*The ROIs included all cortical voxels within a 15 mm radius of the centre. IFG, inferior frontal gyrus; ATC, anterior temporal cortex.*

### Corpus Callosum Size Evaluation

The total CC cross-sectional area was examined in a subgroup of 45 individuals, which included 11 men and 34 women (age range 34–59 years, mean age 47.1 ± 1.1). Among these individuals, 29 were *PICALM AA&AG* carriers (age-range 34–59 years, mean age 46.2 ± 1.5), and 16 were *PICALM GG* carriers (age range 34–59 years, mean age 49.3 ± 1.5) (Table 3).

**TABLE 3:** Demographic and psychometric characteristics of participants with corpus callosum evaluation.

	*PICALM AA&AG*	*PICALM GG*	*p*-values
*N*	29	16	–
Age range	34–59	34–59	–
Age (years)	46.2+1.5	49.3+1.5	0.18
Sex (men/women)	10/19	1/15	<0.01
Education (years)	15.0+0.1	14.8+0.2	0.29
*ApoE E4-/ApoE E4*+	17/12	12/4	0.02
MMSE	29.1+0.1	28.8+0.2	0.13
COWAT (words)	53.0+2.1	45.7+2.4	0.04
LMWT	5.9+0.2	5.6+0.3	0.52
SST errors (number)	0.71+0.1	1.3+0.3	0.053

*Abbreviations are the same as in the Table 1.*

MRI was performed on a 1.5 T Siemens scanner (Magneton Avanto, Siemens, Erlangen, Germany) using a protocol that included a T1-weighted MPRAGE sequence (TR-1940 ms, TE = 3.1 ms; FA = 8; sagittal acquisition with FOV = 25 × 25, matrix 256 × 256 and 1 mm thick slices).

The evaluation of CC size was performed on the mid-sagittal plane of the MRI volumes in accordance with a previously published algorithm. The CC was contoured with automatic calculation of the number of pixels in the selected area (Milovanova et al., 2015).

### Genetic Analysis

Genomic DNA was isolated from peripheral venous blood using a standard phenol-chloroform extraction method or a Qiagen DNA isolation kit (Qiagen, Netherlands). *PICALM* genotyping was performed by PCR followed by restriction fragment length polymorphism (RFLP) analysis as previously described (Ponomareva et al., 2017). *ApoE* genotyping was performed using PCR followed by RFLP analysis. Amplification was performed according to the manufacturer’s instructions using both a Tercyc DNA amplifier (DNA technology, Russia) and a GeneAmp PCR System 9700 Thermal Cycler (Applied Biosystems) as previously described (Ponomareva et al., 2017).

### Statistical Analysis

The significance of the differences in the LLC solutions between the pairs of 16 ROIs in each frequency band across groups of subjects with *PICALM AA&AG* or *PICALM GG* was estimated using the eLORETA independent sample *t*-test, generating *t*-statistics for brain connectivity. For the functional connectivity analysis, we performed 840 tests using eLORETA to examine all connections between the 16 ROIs (120 connections) in seven frequency bands (120 × 7 = 840). For correcting for multiple comparisons, we applied the eLORETA non-parametric randomization method based on the “maximal statistic” (Holmes et al., 1996; Nichols and Holmes, 2002).

We combined the groups with the *PICALM AA* and *PICALM AG* genotypes into a single group because the group with the *PICALM AA* genotype was relatively small (18 subjects) and because the control analysis of genotype (*PICALM AA* vs *PICALM AG*) did not show significant differences in the LLC parameters between the *PICALM AA* and *PICALM AG* groups.

The demographic and psychometric data were tested for a normal distribution with the Wilk–Shapiro test. The demographic and psychometric scores were compared with analysis of variance in cases of a normal distribution or with Mann–Whitney *U*-tests.

Percentage of men/women and *APOE* distribution in the groups of subjects with *PICALM AA&AG* and *PICALM GG* genotypes were compared using the Chi-square test.

To assess the association between functional connectivity and the verbal memory in the groups with different *PICALM* genotypes as well as in the entire sample, we used eLORETA software. The correlation between LLC values of all pairs of ROIs in each frequency band and LWMT scores was computed and the significance level of *r*-values was determined by nonparametric randomization for multiple comparison correction (Nichols and Holmes, 2002). The critical probability threshold was set at *p* < 0.05.

A similar analysis was performed in order to assess the correlation between LLC values and the CC size in the examined groups.

When the correlation did not exceed the corrected critical threshold *p* < 0.05, Pearson correlations were calculated in cases with a normal distribution, and Spearman rank correlations were calculated in other cases. An uncorrected significance level of *p* < 0.05 was considered to indicate a tendency.

To control the influence of age on the results, we performed correlation analysis between the LLC values of all pairs of ROIs in each frequency band and the age of the individuals.

## Results

### Comparison of eLORETA LLC Solutions in Non-demented Individuals With Different *PICALM* Genotypes

**FIGURE 1:**
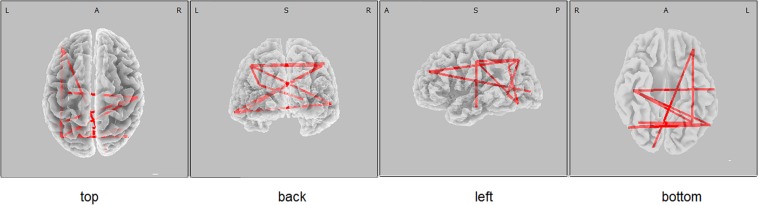
eLORETA wire diagram indicating cortical regions with significantly higher alpha lagged linear connectivity (threshold 2.92, *p* < 0.05) in non-demented carriers of *PICALM AA&AG* genotypes compared with the carriers of *PICALM GG* genotype.

The functional connectivity of the carriers of the *PICALM AA&AG* genotypes, compared with the carriers of the *GG* genotype, showed higher alpha LLCs over several cortical regions (threshold *t* = 2.92, *p* < 0.05) (Figure 1). There were no significant differences in functional connectivity in other frequency bands between the carriers of different *PICALM* genotypes. The differences of LLC characteristics in the individuals with the carriers of *ApoE E4+* and *ApoE E4-* genotypes were not significant.

Control analysis showed that no significant correlation between LLC characteristics and age was observed in the groups of *PICALM AA&AG* and *PICALM GG* genotype carriers as well as in the whole sample.

### Correlation of the LLC Interhemispheric Solutions and the Luria Memory Word Test Scores in Individuals With Different *PICALM* Genotypes

**FIGURE 2:**
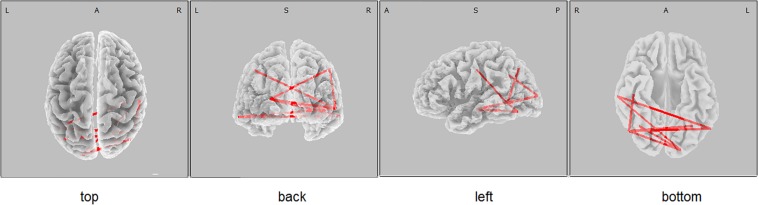
eLORETA wire diagram of significant positive correlation between the lagged linear connectivity (LLC) of alpha cortical sources and the scores of Luria memory word test (LMWR) in the entire sample including individuals with the *PICALM AA&AG* and *PICALM GG* genotypes. The red wires indicate the locations with significant positive correlation.

**FIGURE 3:**
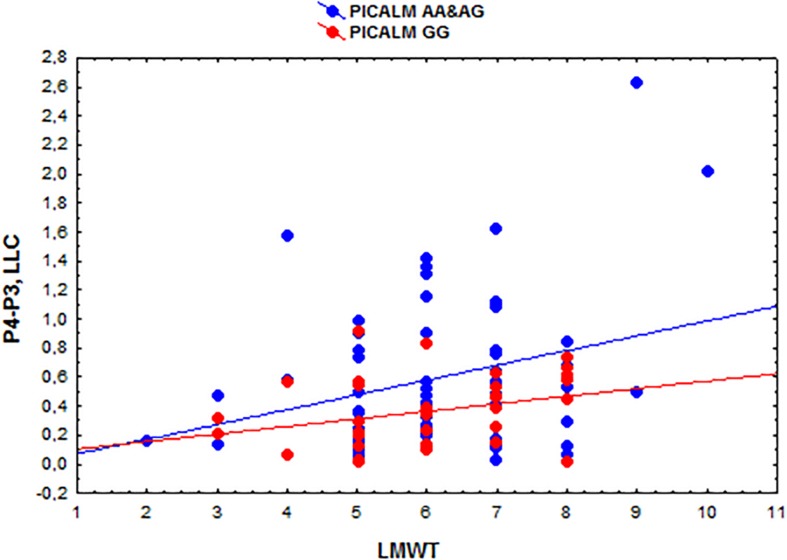
Scatterplots showing the correlations among the interhemispheric lagged linear connectivity (LLC) of alpha cortical sources in the parietal regions (P4-P3) and the scores of Luria memory word test (LMWT) scores in the subjects with the *PICALM AA&AG* and *PICALM GG* genotypes.

The eLORETA correlation analysis (corrected for multiple comparison critical threshold *p* < 0.05) in the entire sample of individuals with different *PICALM* genotypes showed that the LMWT scores had a positive correlation with the LLC solutions in the alpha sources in several area (O1P4 *r* = 0.3; O1T6 *r* = 0.29; O1T4 *r* = 0.29; P3P3 *r* = 0.26; P3T6 *r* = 0.29; P3T4 *r* = 0.27; C4T6 *r* = 0.29; T6T5 *r* = 0.3; T5T4 *r* = 0.29). In the groups with *PICALM AA&AG* as well as with *PICALM GG* genotypes when considered separately correlation did not exceed the corrected critical threshold *p* < 0.05) (Figures 2, 3).

### Correlation of the LLC Interhemispheric Solutions and the CC Size in Individuals With Different *PICALM* Genotypes

The total CC cross-sectional area did not differ between the carriers of the *PICALM AA&AG* and *PICALM GG* genotypes (637.7 +15.1 mm^2^ and 623.0 +20.9 mm^2^ in *PICALM AA&AG* and *PICALM GG*, respectively).

**FIGURE 4:**
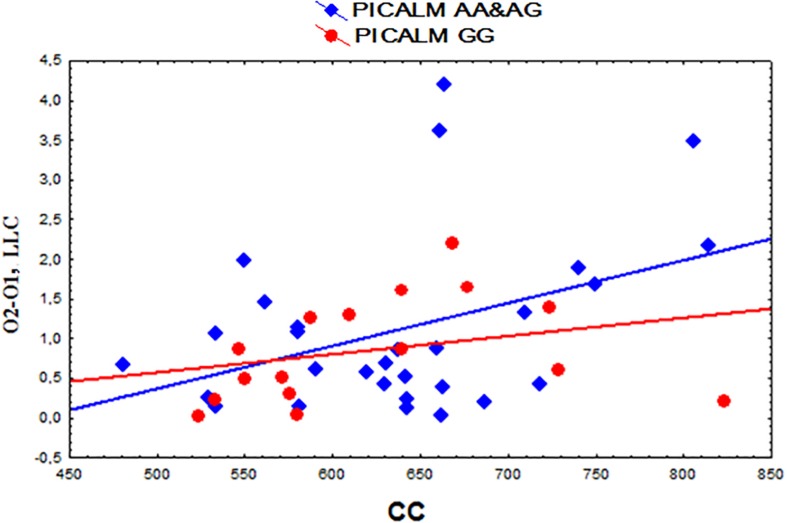
Scatterplots showing the correlation between the interhemispheric lagged linear connectivity (LLC) of alpha cortical sources in occipital regions (O2-O1) and the corpus callosum (CC) size (mm^2^) in the carriers of the different *PICALM* genotypes.

A positive correlation was found between the functional interhemispheric LLC solutions in the occipital alpha sources and the total CC size (*r* = 0.37, *p* = 0.013 uncorrected) (Figure 4). The same correlation was found in the subjects with the *PICALM AA&AG* genotypes as a whole group (*r* = 0.40, *p* = 0.032 uncorrected). In the carriers of the *PICALM GG* genotype, the correlation was not significant *PICALM GG r* = 0.29, *p* = 0.27).

When corrected for multiple comparisons, correlation between the LLC characteristics and the CC size did not exceed the significance threshold *p* < 0.05.

## Discussion

The main findings of this study show that in non-demented adults younger than 60 years of age, the *PICALM* rs3851179 polymorphism (the presence of protective *A* allele vs homozygous AD risk *GG* variant) is associated with characteristics of functional EEG connectivity. We found that the carriers of the *PICALM A* allele (*PICALM AA&AG* genotypes) had higher widespread interhemispheric LLC of alpha sources compared to the carriers of the *GG PICALM* allele. Importantly, the LLC characteristics of EEG connectivity used in the present study are not affected by the electrophysiological artefacts of volume conduction.

The rsEEG connectivity deficit in the carriers of the AD risk variant *PICALM GG* could represent functional alterations in alpha rhythm networks. These alterations may be related to the same factors as the decrease in the hippocampal functional connectivity found in younger carriers of the *PICALM GG* genotype in a fMRI study (Zhang et al., 2015). In elderly subjects, *PICALM GG* genotype is associated with lower hippocampal volume, decreased entorhinal cortex thickness and earlier cognitive decline (Biffi et al., 2010; Furney et al., 2011; Sweet et al., 2012).

The hippocampus is an important node of the DMN that modulates EEG alpha activity. The DMN regions, and the hippocampus in particular, are preferentially vulnerable to degenerative processes that are accompanied by progressive decreases in LLC of alpha sources (Babiloni et al., 2018). In MCI and prodromal AD, alpha disconnection in brain networks is related to progressive cognitive impairment. The alterations in connectivity may be influenced by the accumulation of amyloid and tau deposition, synaptic disruption and impaired axonal integrity (Canuet et al., 2015; Ishii et al., 2017).

Our previous studies have shown that the *PICALM* genotype is associated with characteristics of rsEEG beta power and with latency of P300 cognitive evoked potentials. The carriers of the *PICALM GG* genotype have a more pronounced increase in ERP P300 latency and higher rsEEG beta power compared to the carriers of the *PICALM A* allele (Ponomareva et al., 2017; Ponomareva et al., 2018). These effects were more pronounced in non-demented subjects older than 50 years of age, whereas the difference in LLC characteristics of alpha sources was observed in younger subjects.

The mechanisms underlying the association between the *PICALM* genotype and alpha functional connectivity are not completely understood. Previous studies have revealed that the *PICALM* rs3851179 *A* allele modestly increases *PICALM* expression (Parikh et al., 2014). The PICALM protein is involved in CME. An abnormal level of *PICALM* may be related to dysfunction of endocytosis. *PICALM* modulates synaptic function by facilitating neurotransmitter delivery and by influencing the cell surface level of the glutamate receptor subunit GluR2 (Harel et al., 2008; Harel et al., 2011). *PICALM* also influences the pathways that directly contribute to the development of the pathological hallmarks of AD, including the accumulation of Aβ and hyperphosphorylated tau proteins in the brain (Xiao et al., 2012; Ando et al., 2013; Moreau et al., 2014; Xu et al., 2015; Zhao et al., 2015). However, the exact mechanisms by which the *PICALM* rs3851179 polymorphism can contribute to the interhemispheric functional connectivity of rsEEG alpha sources in younger subjects requires further investigation.

According to the recently proposed diagnostic criteria of AD, the preclinical AD stage is characterized by abnormal deposition of amyloid-β in the brain, as well as by a reduction of Aβ42 and increased phospho-tau in the cerebrospinal fluid (CSF) (Dubois et al., 2014). The abnormal deposition of Aβ in the brain can be revealed by standard uptake value ratio of amyloid sensitive positron emission tomography (PET) tracers, such as 18F-florbetapir, florbetaben, flutemetamol or 11C-PIB and tau PET load. There are promising EEG candidate biomarkers of preclinical AD including reduction of functional cortical connectivity (Babiloni et al., 2018). Recent studies have shown an association between EEG alpha rhythm alterations and AD risk variant in the *ApoE* gene in AD, MCI patients and even in healthy adults (Lizio et al., 2011, review; Ponomareva et al., 2008, 2012).

The results obtained in the present study suggest several possibilities. First, the examined *PICALM GG* genotype carriers with reduced LLC may have a preclinical stage of AD. As we were not able to examine Aβ42, phospho-tau in the CSF, or amyloid and tau PET load in the present study, it is not possible to definitively conclude if this is the case. Second, the reduction of EEG connectivity in these individuals may be a risk factor for AD development without necessarily being a marker of preclinical stage of AD. Finally, the higher functional connectivity in the carriers of the protective *PICALM A* allele may underlie higher neurophysiological reserve. As was shown in prior studies, higher neurophysiological reserve ensures better coordination of neural activity across neural networks. This neurophysiological reserve may contribute to better cognitive performance and/or greater resilience against pathological processes that accompany the development of the disease (Babiloni et al., 2018). These possibilities could be verified by longitudinal studies of disease incidence in the examined individuals, as well as studies examining the standard biomarkers of preclinical AD in these individuals.

A significant but modest positive correlation (0.01 < *p* < 0.05 corrected for multiple comparison) was found between the alpha LLCs and LWMT scores in the whole sample. The correlation under a more stringent criteria *p* < 0.01 has not reached statistical significant value after multiple comparison correction. The results indicate that an expected false discovery rate (FDR) of *r*-values is less than 5%. Previous studies also demonstrated that memory characteristics are related to brain network connectivity (Fokin et al., 2019; Vecchio et al., 2019). There were no significant differences in the LMWT scores or in the other cognitive tests that we applied in the carriers of *PICALM AA&AG* and *PICALM GG* genotypes. Detailed cognitive assessment was beyond the scope of this study. Higher neurophysiological reserve may not necessarily correspond to higher cognitive scores but instead to better resilience against cognitive decline in aging. The association between the *PICALM A* allele and better composite cognitive scores was found in older men without dementia (Mengel-From et al., 2011).

Previous studies have demonstrated a correlation between alpha connectivity and the size of the CC, the major brain commissure, and its subregions. The CC, plays a fundamental role in integrating information between hemispheres and maintaining functional connectivity (Hinkley et al., 2012; Roland et al., 2017). Callosal atrophy has been shown to be related to the degree of cognitive decline in patients with MCI and AD. The degree of callosal atrophy may be used as a biomarker for cognitive deficits even before these deficits meet the diagnostic criteria for AD (Wang et al., 2015, review).

Our results show that the correlation between the LLC characteristics and the CC size was only significant if correction for multiple comparisons was not performed. Therefore, we can only consider the relation of the LLC characteristic of functional interhemispheric connectivity with the size of the main interhemispheric commissure CC to be a tendency rather than a definite dependence. We did not find a significant association between the CC size and the *PICALM* genotype. It is possible that other structural and functional mechanisms may modulate alpha LLC and its association with *PICALM* genotype.

The limitations of the present study include the relatively small number of participants (104 subjects), especially the carriers of the *PICALM AA* genotype (18 subjects), which which was insufficient to examine differences of the LLC parameters in the this group and made us combine the carriers of the *PICALM AA* and *PICALM AG* genotypes into a single group. However, the LLC differences between the groups of subjects carrying the *PICALM GG* and *PICALM AA&AG* genotypes were found using eLORETA independent sample *t*-test with eLORETA non-parametric randomization method based on “maximal statistic” for correcting multiple comparisons (Holmes et al., 1996; Nichols and Holmes, 2002).

Because the evaluation of CC size was performed only on a subset of the participants, and the subgroups with the *PICALM AA&AG* and *PICALM GG* genotypes were not gender-matched, the results on the lack of association of *PICALM* genotype and CC size are preliminary. Nevertheless, the finding on the moderate dependence of the LLC characteristics on the CC size on the entire sample can contribute to an understanding of the structural basis of functional connectivity.

Another limitation is the small number of electrodes. Sixteen scalp electrodes (e.g., 10–20 system) were used for EEG recording, while an optimal EEG recording may use 64–128 electrodes. However, 16 electrodes may be still acceptable for rsEEG with diffuse cortical neural synchronization. Such a distributed pattern may not require the high spatial sampling of EEG activity that is necessary to study event-related potentials (Babiloni et al., 2018). Further studies need to be carried out to validate the association between the *PICALM* genotype and EEG connectivity.

In summary, in this study, we found that non-demented adults younger than 60 years of age carrying the *PICALM* rs3851179 *A* allele had higher interhemispheric functional connectivity of rsEEG alpha sources compared to the carriers of the *PICALM GG* genotype. These results suggest that the *PICALM* rs3851179 *A* allele renders protection against cognitive decline by facilitating neurophysiological reserve capacities in non-demented adults. In contrast, lower functional connectivity in the carriers of the AD risk *PICALM GG* genotype suggests early functional alterations in alpha rhythm networks.

## Data Availability Statement

The raw data supporting the conclusions of this article will be made available by the authors, without undue reservation, to any qualified researcher.

## Ethics Statement

The studies involving human participants were reviewed and approved by the local Ethics Committees of Research Center of Neurology, Moscow, Russia and Vavilov Institute of General Genetics, RAS, Moscow, Russia. The patients/participants provided their written informed consent to participate in this study.

## Author Contributions

NP, ER, and SI contributed to the conceptualization of this manuscript. NP, ER, and SI designed the research. NP, TA, MP, and DM performed the research. NP, VF, AM, ER, MK, and RK contributed the data analysis. NP wrote the manuscript.

## Conflict of Interest

The authors declare that the research was conducted in the absence of any commercial or financial relationships that could be construed as a potential conflict of interest.

## Footnotes

1 http://www.AlzGene.org
